# Revisiting berberine for the prevention and treatment of *Fusobacterium nucleatum*-induced colorectal cancer from a dynamic perspective

**DOI:** 10.3389/fmicb.2025.1637272

**Published:** 2025-09-11

**Authors:** Dongming Bi, Xue Yang, Jiangyan Yong, Ju Huang, Zhihao Liu, Rui Gong

**Affiliations:** 1 Department of Laboratory Medicine, Hospital of Chengdu University of Traditional Chinese Medicine, Chengdu, Sichuan, China; 2 Department of Laboratory Medicine, Deyang Hospital Affiliated Hospital of Chengdu University of Traditional Chinese Medicine, Deyang, Sichuan, China; 3 Department of Respiratory Medicine, Hospital of Chengdu University of Traditional Chinese Medicine, Chengdu, Sichuan, China

**Keywords:** *Fusobacterium nucleatum*, colorectal cancer, berberine, adenoma, adenocarcinoma

## Abstract

Colorectal Cancer (CRC), a common malignancy, often arises from adenomatous precursors. In the adenoma-carcinoma progression of CRC, *Fusobacterium nucleatum* (Fn) plays an important driving role. Therefore, the discovery of new drugs targeting Fn-induced disease progression is crucial for the prevention and treatment of CRC. Berberine (BBR), which has a relatively broad spectrum of antitumor activity, has received increasing attention in recent years. In this study, we summarize BBR's regulatory effects on the different stages of intestinal adenoma-carcinoma transformation induced by Fn and its anti-tumor mechanisms in the occurrence and development of CRC for the first time. Firstly, BBR can prevent the migration and intestinal colonization of Fn and regulate Fn-induced microbiota imbalance. Secondly, in the pre-cancerous lesion stage, BBR can attenuates Fn-mediated inflammation, inhibit abnormal crypt foci, and reverse adenoma progression. In addition, BBR can suppresses established CRC by inhibiting cell proliferation, invasion, metastasis, immune escape and drug resistance. For the classic pathogenic model of Fn-mediated CRC, the therapeutic effect of BBR is dynamic and comprehensive from pathogenic factors to pathological products. Among them, E-cadherin, Wnt/β-catenin, JAK/STAT and MAPK/ERK signaling pathways may be key to BBR's prevention of Fn-induced CRC.

## 1 Introduction

Colorectal Cancer (CRC) ranks among the top three malignancies worldwide in terms of both incidence and mortality, representing a major disease that severely threatens human health (Bray et al., 2024). Epidemiological data indicate that approximately 154,000 new CRC cases and 53,000 deaths are projected to occur in the United States in 2025 (Siegel et al., 2025). CRC has a hidden onset and atypical early symptoms, and most patients are diagnosed in the middle and late stages. Therefore, prevention of the etiology of CRC, as well as early screening and intervention of the disease, are of great significance in prolonging patient survival and improving quality of life. Studies have shown that 60%-70% of CRC is derived from adenomas, a recognized precancerous lesion that typically takes 5-10 years to develop (Shaukat et al., 2021; Simon, 2016).

With further research, the influence of chronic inflammation and intestinal microbiota on the adenoma-carcinoma progression of CRC has gradually been recognized (Sepich-Poore et al., 2021). Inflammation is a high-risk factor for CRC, while dysbiosis of the intestinal microbiota is closely related to the recurrence of inflammation (Tlaskalova-Hogenova et al., 2004). In fact, there are thousands of microorganisms colonized in the human intestine, maintaining normal physiological functions and resisting the invasion of pathogens (Tremaroli and Bäckhed, 2012). However, the ecological imbalance of bacteria, fungi, and even viruses in the intestine will disrupt the delicate balance between them and their host, leading to immune system damage and disease occurrence (Gibiino et al., 2017).

Compared with healthy individuals, the intestinal microbiota in the intestinal lumen and mucosa of CRC patients undergoes changes. The reduction of beneficial bacteria and the increase of opportunistic pathogens may promote the transformation of benign adenomas into malignant adenocarcinomas (Wang et al., 2012; Zhang et al., 2018). Among them, *Fusobacterium nucleatum* (Fn) is an opportunistic anaerobic symbiotic bacterium in the human oral cavity, which is relatively rare in healthy intestines (Warren et al., 2013). However, enriched Fn has been detected in the tissues or rectal swabs of CRC patients, and the accumulation of Fn is associated with the development of adenoma-carcinoma progression (Proença et al., 2018; Flanagan et al., 2014). The colonization of Fn in the intestine can lead to defects in the epithelial barrier (disruption of tight junctions and cell-to-cell contacts, loss of epithelial polarity and mucus layer) and local dysplasia (Grivennikov et al., 2012), thereby promoting the occurrence of CRC in the body (Yu et al., 2015). Therefore, the discovery of new drugs targeting Fn-induced disease progression is crucial for the prevention and treatment of CRC.

**Figure 1:**
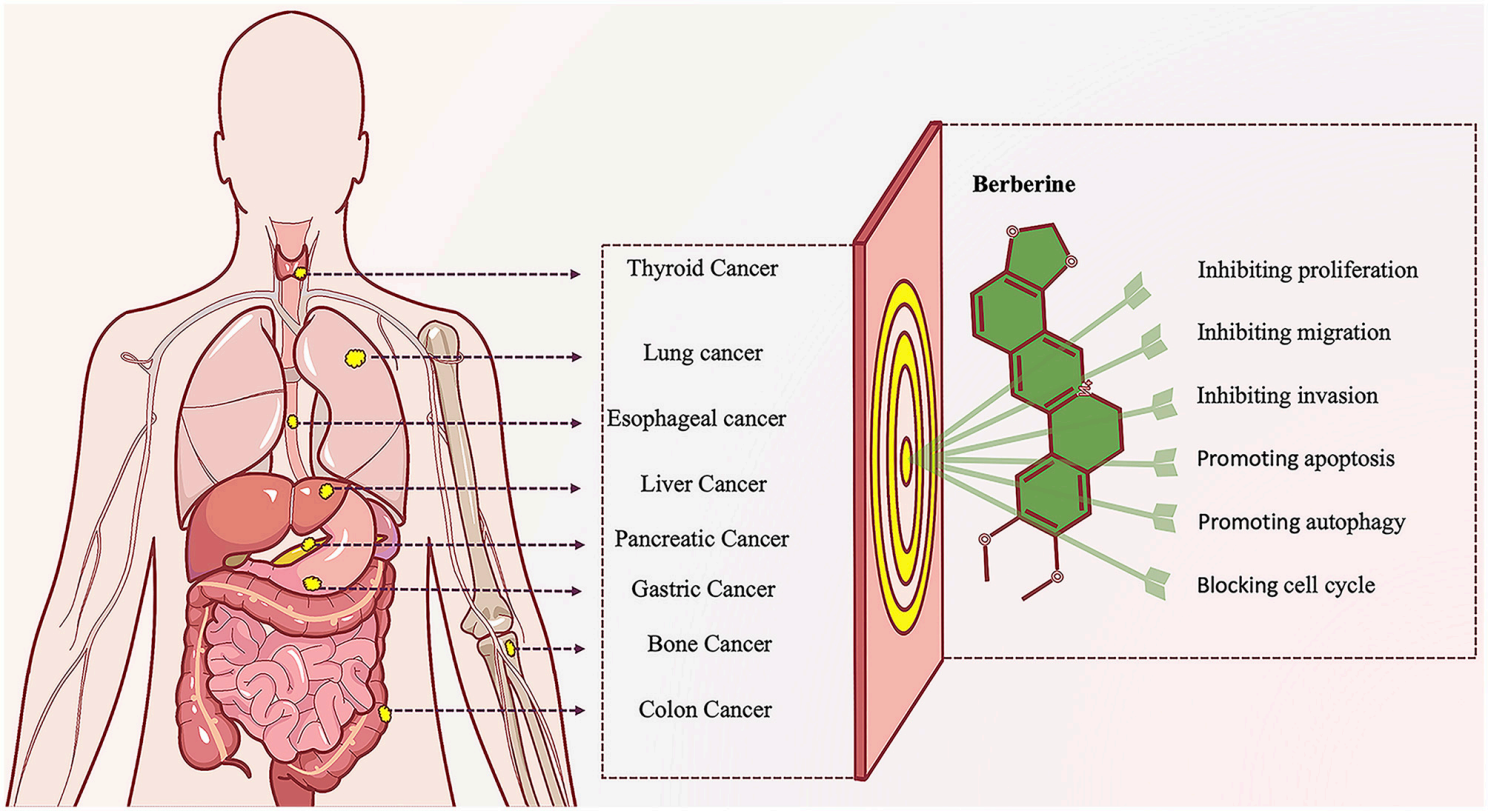
Diagram of anti-tumor effect of Berberine. Berberine, a naturally occurring alkaloid with diverse biological activities, has demonstrated remarkable potential in anti-tumor research. This compound effectively suppresses the initiation and progression of various malignancies through complex and interconnected molecular mechanisms and signaling pathways. Its multifaceted actions precisely target multiple critical pathways, such as inhibiting abnormal tumor cell proliferation, migration, and invasion; promoting tumor cell apoptosis and autophagic cell death; and arresting aberrant cell cycle progression. This orchestrated multi-target synergy establishes berberine as a promising translational candidate in contemporary anti-tumor drug discovery.

The treatment of CRC mainly includes surgical treatment, radiotherapy, chemotherapy, immunotherapy, etc (Argiles et al., 2020). However, due to adverse reactions, drug resistance, and high recurrence rates (Argiles et al., 2020), an increasing number of people are turning to natural products and their derivatives, hoping to use their multi-target mechanisms and complex pharmacological activities to benefit patients. Berberine (BBR), a quaternary ammonium isoquinoline alkaloid, has received attention for its relatively broad spectrum of antitumor activity. Studies have shown that BBR has inhibitory effects on various tumors including CRC (Figure 1) (Wang Y. et al., 2020; Rauf et al., 2021). BBR not only delays the progression of CRC but also has a therapeutic effect on the intestinal adenoma-carcinoma process induced by Fn.

In this review, we summarize the sequential effects of BBR on the Fn-induced intestinal adenoma-carcinoma cascade from a dynamic perspective and its regulatory mechanism in the occurrence of CRC for the first time. This study is expected to provide preclinical evidence for the use of BBR in the prevention and treatment of CRC, and the development of related new drugs will further reduce the incidence and mortality of CRC.

## 2 Intestinal adenoma-carcinoma transformation induced by Fn

**Figure 2:**
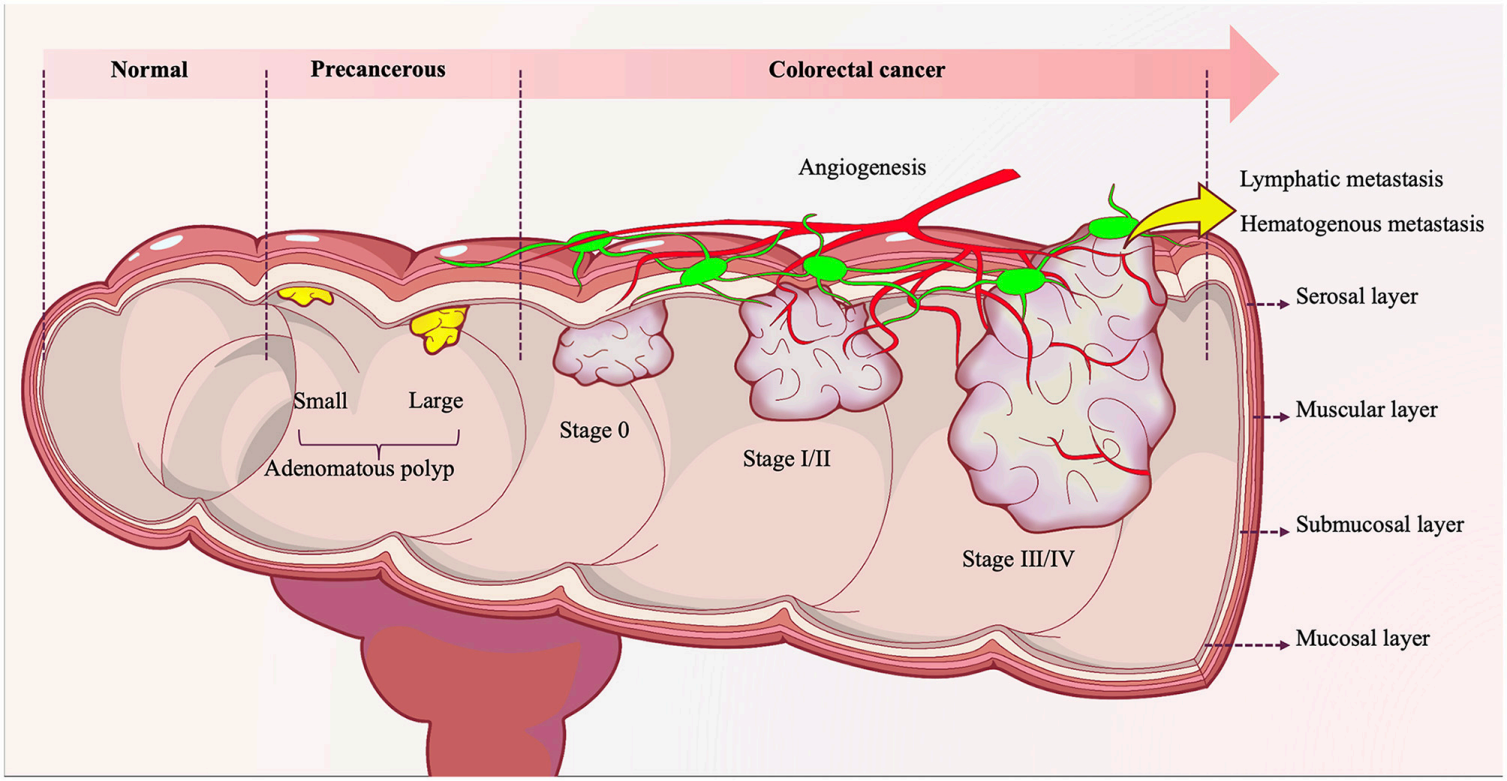
The evolution of colorectal adenoma-carcinoma progression. Under the long-term synergistic effects of both intrinsic and extrinsic pathogenic factors, the normal colonic mucosal epithelium initially undergoes atypical hyperplasia, characterized by abnormally accelerated proliferation rates, architectural disorganization, and loss of cellular polarity. With progressive injury accumulation, the mucosal epithelium evolves into precancerous lesions such as tubular adenomas, villous adenomas, or tubulovillous adenomas. During the adenomatous stage, accumulating genetic mutations drive progression from low-grade to high-grade intraepithelial neoplasia, marked by significantly enhanced cytological atypia and emerging invasive potential. Upon breaching the basement membrane and infiltrating the submucosa and deeper tissues—concomitant with acquired capabilities for vascular and lymphatic invasion—the adenomatous lesion ultimately completes its malignant transformation into colonic adenocarcinoma.

The formation of CRC is not a sudden event, but a process that goes through normal mucosa, mucosal epithelial hyperplasia, adenomatous polyps, gradually increasing adenomatous polyps, colorectal cancer (Figure 2) (Lucas et al., 2017). In this process, Fn plays an important role. In fact, the causal relationship between microorganisms and tumors is not uncommon, such as Helicobacter pylori and gastric cancer, human papillomavirus and cervical cancer, hepatitis B virus and liver cancer. In the early stage of the intestinal adenoma-carcinoma cascade, Fn can be detected to accumulate in adenomas (Kostic et al., 2013). Furthermore, Fn can increase the count of Aberrant Crypt Foci (ACF), adenomas and adenocarcinomas, and promote the occurrence and progression of intestinal tumors (Kostic et al., 2013). Studies have shown that more than 40% of CRC patients can detect the same Fn in their intestines and oral cavities (Komiya et al., 2019). Fn can stably adhere and invade endothelial cells, epithelial cells and tumor stem cells (CSCs) through multiple pathways, causing intestinal and systemic spread (Han et al., 2004). This migration process involves the participation of actin, microtubules, pathways, protein synthesis, and energy metabolism (Han et al., 2000).

With the accumulation of Fn, the balance of intestinal microbiota is disrupted, triggering innate immune responses and activating signaling pathways such as janus kinase/signal transducer and activator of transcription (JAK/STAT), mitogen-activated protein kinase/extracellular signal-regulated kinase (MAPK/ERK), and nuclear factor kappa-B (NF-κB). At the same time, it induces the secretion of interleukin-8 (IL-8), IL-21, IL-22, IL-24, IL-31 and CD40 (Kostic et al., 2013; Yu et al., 2015; Han et al., 2000; Cavallucci et al., 2022). Stem cells infected by Fn can be selectively recruited to the submucosal layer and migrate to the mucosal layer, increasing the susceptibility of intestinal tumors by activating the classical Wnt/β-catenin/TGIF signaling pathways (Lin et al., 2020). Further research has shown that Fn-derived formates can also increase the CSCs and self-renewal ability of CRC by triggering AhR signaling and Th17 cell expansion (Ternes et al., 2022). Therefore, effectively inhibiting the immune response caused by Fn and reversing epithelial dysplasia in the pre-cancerous stage are potential research directions in the field of CRC prevention and treatment.

Pharmacological studies have shown that BBR can exert anti-Fn effects while preventing and treating CRC through diverse mechanisms or pathways.

## 3 Stage 1: delaying the Fn

### 3.1 BBR inhibits the migration of Fn

Fn naturally resides in the oral and other mucosal areas of both humans and animals, and plays an essential role in the formation of dental biofilm (Brennan and Garrett, 2019). Fn is considered both a common commensal bacterium and an opportunistic pathogen, and has been associated with various oral diseases such as acute appendicitis, amniotic fluid infections, liver abscesses, and osteomyelitis (Brennan and Garrett, 2019). Targeted sequencing results have shown that Fn in the gut originates from the oral cavity, and adheres to intestinal tissue early in the development of CRC (Komiya et al., 2019). In the APC^Min/+^ mouse model, oral administration of Fn can accelerate CRC occurrence in the absence of intestinal inflammation (Kostic et al., 2013). In addition to the primary oral-digestive tract pathway (Kostic et al., 2013), the enrichment of Fn in CRC tissue may also be targeted to cancer colonies via a bloodborne pathway, such as transient bacteremia caused by dental surgery or periodontitis (Bullman et al., 2017; Yang and Shamsuddin, 1996). Studies have confirmed that intravenous injection of Fn can target mouse tumor tissue in a Fap2-dependent manner (Abed et al., 2016). BBR has a strong inhibitory effect on Fn (Fukamachi et al., 2015). On one hand, BBR can directly kill Fn in the oral cavity, intercepting bacteria at the upstream end of the digestive tract to prevent migration to the intestine (Xie et al., 2012). On the other hand, because BBR is difficult to absorb after oral administration, it mainly stays in the intestine and interacts with the microbiota (Feng et al., 2018) to combat Fn located in the mucus layer and intestinal crypts (McCoy et al., 2013).

### 3.2 BBR prevents the intestinal colonization of Fn

The adhesion and colonization of bacteria are prerequisites for their functional activity. Upon reaching the intestine, Fn secretes adhesin FadA with starch-like properties. FadA is divided into intact pre-FadA and secreted mature FadA (mFadA), with pre-FadA located in the inner membrane and mFadA secreted outside the bacteria (Xu et al., 2007). As adenomas progress to adenocarcinomas, the expression levels of FadA in tissues gradually increase (Rubinstein et al., 2013). By binding to E-Cadherin on intestinal epithelial cells, FadA can mediate further attachment and invasion of Fn into the host (Han et al., 2005). About 70% of primary CRC specimens show predominantly membranous expression of E-Cadherin (Palaghia et al., 2016). Downregulation of E-cadherin expression significantly inhibits Fn attachment and invasion of HCT116 cells (Rubinstein et al., 2013). In addition, the adhesin Fap2 on the surface of Fn can recognize Gal-Gal-NAc overexpressed in CRC and CEACAM1 in the carcinoembryonic antigen family, thereby targeting the intestine (Abed et al., 2016; Coppenhagen-Glazer et al., 2015). BBR can effectively block bacterial adhesion to the intestine (Sun et al., 1988), maintain intestinal villus integrity and normal intestinal epithelial cell structure (Izadparast et al., 2022). At the same time, BBR can increase the expression of intestinal tight junction proteins (ZO-1 and occludin), downregulate the NF-κB and myosin light chain kinase pathways to maintain epithelial structure, and prevent bacterial penetration of the intestinal mucosal barrier (Deng et al., 2022; Gu et al., 2011).

### 3.3 BBR regulates the intestinal microbiota imbalance caused by Fn

The changes in the gut microbiota are associated with the earliest stages of tumor development (Sanapareddy et al., 2012). As normal colorectal tissue progresses to adenomas and CRC, the balance of the gut microbiota is disrupted, with an increase in opportunistic pathogens and a decrease in butyrate-producing bacteria (Chen et al., 2013). Meanwhile, the proportion of Fn gradually increases in colorectal adenoma-carcinoma transition, playing a promoting role (Yu et al., 2015). The gut colonization of Fn can significantly alter the microbial structure of the intestinal lumen by increasing tenericutes and verrucomicrobia (Yu et al., 2015). BBR can reverse the imbalance of gut microbiota caused by Fn colonization, reduce opportunistic pathogens such as tenericutes and verrucomicrobia (Yu et al., 2015), and increase the relative abundance of beneficial gut microbiota (Xie et al., 2011). In addition, obesity is an important risk factor for CRC (Mana et al., 2021). For high-fat diet mice, BBR may reduce the degradation of dietary polysaccharides and calorie intake by regulating the intestinal microbiota, thereby activating the expression of related genes for Fasting-induced adipocyte factor and mitochondrial energy metabolism in visceral adipose tissue, which helps to play an anti-obesity role (Xie et al., 2011).

**Figure 3:**
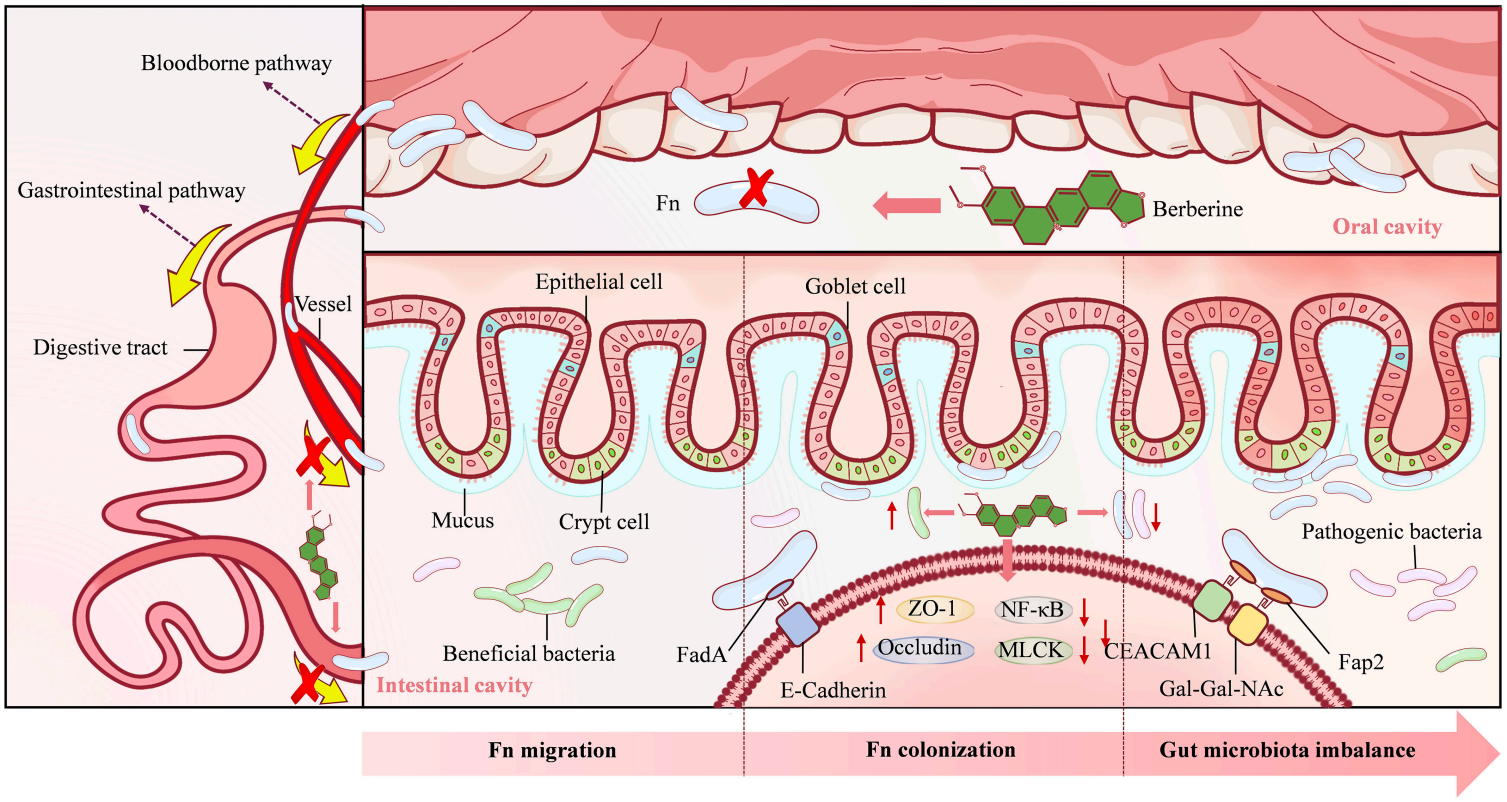
The mechanism underlying Berberine action in stage 1. Berberine can directly kill *Fusobacterium nucleatum* (Fn) in the oral cavity, prevent Fn from migrating and colonizing in the intestine, and regulate the reduction of beneficial bacteria and the proliferation of pathogenic bacteria caused by Fn.

In summary, BBR effectively impedes the migration and colonization of Fn within the intestine and suppresses Fn-induced dysbiosis through its multi-layered synergistic actions: interception at the source, intestinal clearance, barrier reinforcement, and microbiota modulation. Consequently, BBR exerts a crucial preventive intervention during the early stages of CRC development (Figure 3).

## 4 Stage 2: delaying the Fn-induced intestinal adenoma-carcinoma transformation

### 4.1 BBR slows down intestinal inflammation

Under normal circumstances, the microbiota in the gut is separated from the epithelial tissue by a dense layer of mucus. The presence of this mucus layer allows the body to tolerate foreign antigens, thereby limiting inflammatory reactions. However, with the invasion of Fn into the gut mucus layer, the biofilm it forms can promote chronic mucosal inflammation (Dejea et al., 2014). Studies have found that in the Apc^Min/+^ mouse model of intestinal adenomas exposed to Fn, tumor-infiltrating immune cells are selectively recruited to create a pro-inflammatory microenvironment and CRC progression (Kostic et al., 2013). Fn colonization can stimulate the secretion of immune cell cytokines such as IL-21, IL-22, IL-23, IL-31, and CD40L (Yu et al., 2015). The elevation of these cytokines can regulate intestinal barrier function through multiple pathways and promote cell proliferation and migration, which are closely associated with inflammation-related CRC occurrence (Wang and Fang, 2023). In addition, Fn in the gut may activate the JAK/STAT and MAPK/ERK pathways, inhibit anti-tumor immunity, and play an important role in CRC progression (Fang and Richardson, 2005; Yu et al., 2009). Experiments have found that BBR can reverse the increased secretion of immune cell cytokines mediated by Fn (Yu et al., 2015). At the same time, BBR treatment significantly reduced the expression of p-signal transducer and activator of transcription 3 (p-STAT3), p-STAT5, and p-ERK1/2 in mice, blocking the activation of the JAK/STAT and MAPK/ERK pathways induced by Fn (Yu et al., 2015).

### 4.2 BBR inhibits aberrant crypt foci

ACF is a cluster of aberrant glandular structures within the colonic mucosa that forms prior to the development of colon polyps. ACF is considered one of the smallest and earliest histopathological phenomena observable under a microscope during the process of CRC formation and is increasingly recognized as an early indicator of carcinogenesis (Roncucci et al., 2000). The colonization of Fn in the intestine can promote the formation of ACF, which affects the progression of CRC (Wang and Fang, 2023). Studies have shown that following inoculation with Fn during the neonatal period, Apc^Min/+^ mice exhibit enhanced expression of IL-17A, an increase in the number of intestinal ACF, and subsequent acceleration of CRC development (Brennan et al., 2021; Yu et al., 2015). Additionally, the bacterial biofilm formed by Fn covers the surface of the colon and promotes proliferation of crypt epithelial cells by activating STAT3 (Dejea et al., 2014). Fn can upregulate the Wnt/β-catenin signaling pathway by binding to E-cadherin, leading to overexpression of oncogenes (Rubinstein et al., 2019). Overactivation of the Wnt/β-catenin signaling pathway can disrupt the balance between cell proliferation and differentiation, maintain stem cell-like phenotypes in colonic crypt cells, and cause malignant transformation (MacLeod, 2013). BBR can reduce the formation of colonic ACF by inhibiting Cyclooxygenase-2 (COX-2) activity (Fukutake et al., 1998). Moreover, BBR can inactivate Wnt/β-catenin protein signaling and decrease the number of ACF, thus reducing the incidence of CRC (Wu et al., 2012).

### 4.3 BBR reverses adenoma-carcinoma progression

Inflammation is a driving factor in the development of colorectal adenoma (Yan et al., 2022; Huang et al., 2019). Compared to normal intestinal mucosa, Fn is more abundant in adenoma tissue, and its species abundance is significantly positively correlated with local inflammation (McCoy et al., 2013). The increased number of Fn leads to elevated expression levels of cytokines IL-6, IL-10, IL-12, IL-17, and tumor necrosis factor-α (TNF-α), and the presence of mucosal inflammation may contribute to adenoma progression (McCoy et al., 2013). In addition, diet is one of the most important environmental factors in the progression from colorectal adenoma to CRC (Chen et al., 2013). Researchers have found that an inflammatory diet rich in red and processed meats, refined grains, and sugars is associated with an increased risk of Fn-positive CRC (Mehta et al., 2017). BBR can delay the recurrence and transformation of colorectal adenomas into cancer (Yan et al., 2022; Chen Y. X. et al., 2020). By inhibiting the Wnt/β-catenin protein signaling pathway, BBR can significantly reduce intestinal polyp growth in Apc^Min/+^ mice and patients with familial adenomatous polyposis, while also inhibiting the expression of cyclin D1 in polyp samples (Zhang et al., 2013). Additionally, BBR can effectively inhibit the expression of pro-inflammatory cytokines, delay the increase in serum lipopolysaccharide-binding protein, monocyte chemoattractant protein-1, and leptin levels in high-fat-fed rats, and correct the decrease in adiponectin levels after adjusting for body fat, thereby reducing food intake to prevent obesity (Zhang et al., 2012).

**Figure 4:**
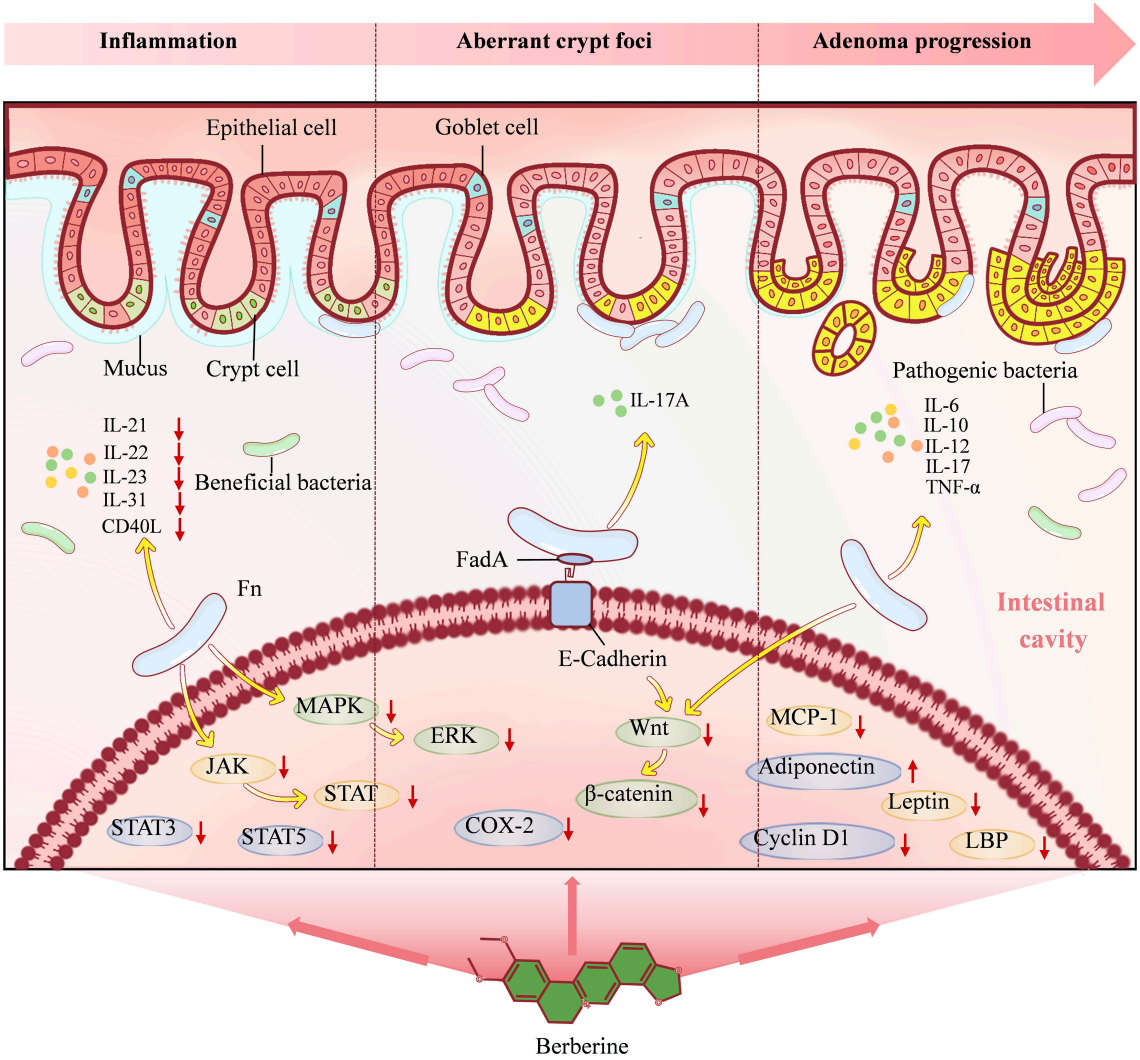
The mechanism underlying Berberine action in stage 2. Berberine alleviates intestinal inflammation, inhibits aberrant crypt foci and reverses adenoma progression through multiple pathways, such as reducing the secretion of immune cytokines, blocking the JAK/STAT, MAPK/ERK and Wnt/β-catenin signaling pathways induced by Fn.

Collectively, BBR establishes a triple defensive strategy comprising the suppression of the pro-inflammatory microenvironment, blockade of early neoplastic marker (ACF) formation, and delay of adenoma progression. This strategy effectively disrupts the Fn-driven intestinal inflammatory cascade and oncogenic signaling pathways (notably the JAK/STAT, MAPK/ERK, and Wnt/β-catenin pathways), thereby providing potent intervention at the critical stage of the colorectal adenoma-carcinoma transition (Figure 4).

## 5 Stage 3: delaying the Fn-associated CRC progression

### 5.1 BBR inhibits the proliferation and cell cycle progression of CRC cells

**Figure 5:**
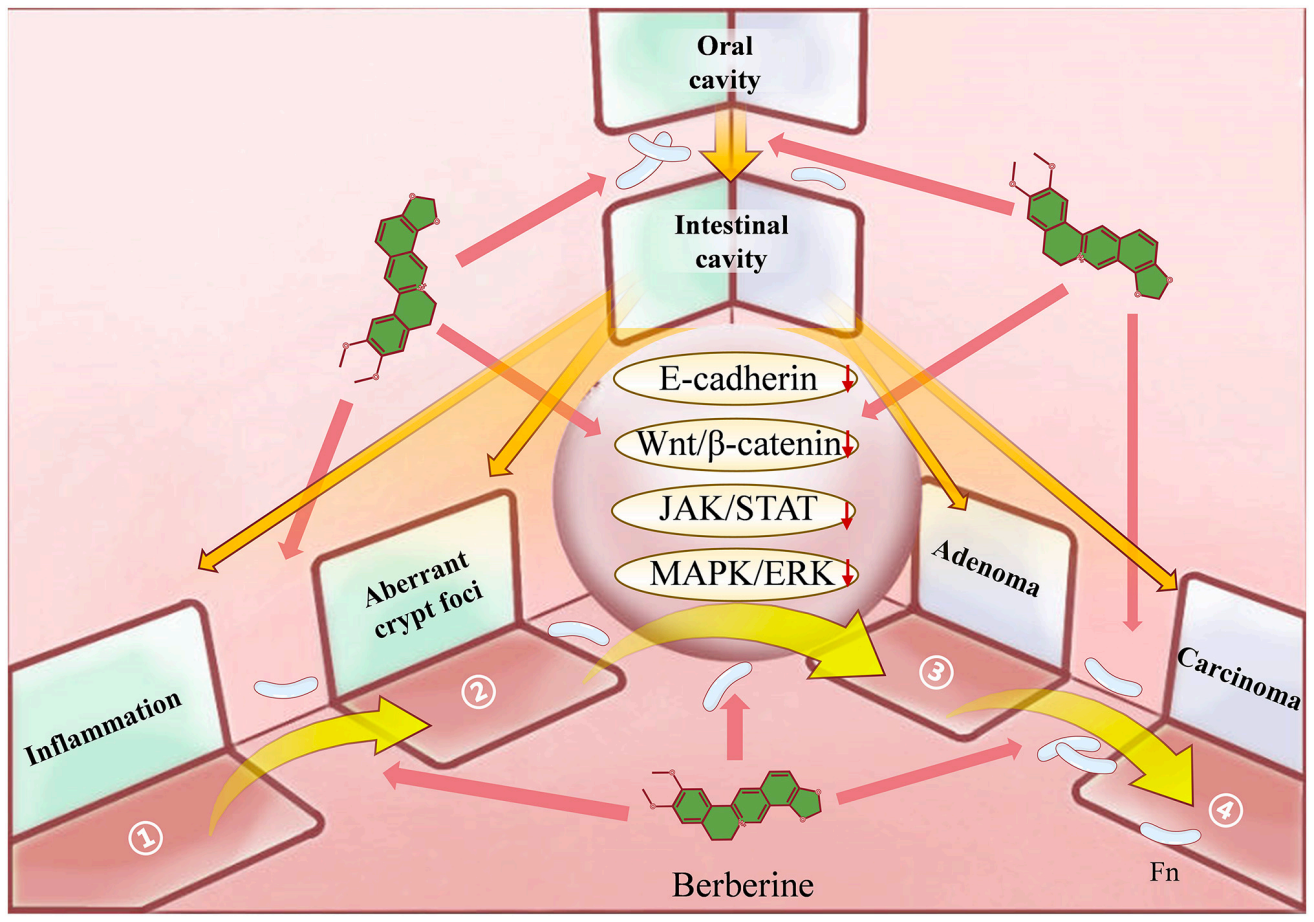
The 3D spatial mechanistic network underlying Berberine's suppression of *Fusobacterium nucleatum* (Fn)-induced colorectal adenoma-to-carcinoma transformation. Against the established Fn-mediated pathogenic model of Colorectal Cancer (CRC), Berberine exerts a comprehensive therapeutic effect spanning from pathogenic initiation to pathological manifestations: It prevents the oral-gut translocation and colonization of Fn and modulates Fn-induced gut dysbiosis. During the precancerous stage, BBR alleviates Fn-driven intestinal inflammation, suppresses aberrant crypt foci, and reverses adenoma progression. Furthermore, BBR impedes the malignant progression of CRC by inhibiting cancer cell proliferation, invasion, metastasis, immune evasion, and drug resistance.

In CRC, Fn infection can activate the toll-like receptor 4 (TLR4)/myeloid differentiation factor 88 (MyD88)/NF-κB signaling pathway, upregulate IL-17F, IL-21, IL-22 and macrophage inflammatory protein-3 alpha (MIP-3a) to promote tumor cell proliferation (Figure 5) (Yang et al., 2017). FadA secreted by Fn not only facilitates adhesion and movement but also acts as a virulence factor (Han et al., 2005). FadA regulates E-cadherin and activates the Wnt/β-catenin signaling pathway, leading to overexpression of Wnt genes, transcription factors, c-Myc oncogenes, inflammatory genes, and CCND1, which stimulate CRC cell growth (Han, 2015; Rubinstein et al., 2013). In addition, in recent years, membrane-associated protein (Annexin-A1) (Guo et al., 2013), a widely distributed calcium-dependent phospholipid-binding protein, has been found to act as a regulator of Wnt/β-catenin and a key growth stimulator of CRC (Rubinstein et al., 2019). Fn selectively stimulates the growth of CRC cells by activating Annexin-A1 (Guo et al., 2013). BBR effectively inhibits the proliferation of CRC cells in a concentration-dependent manner (Wu et al., 2012). By inhibiting the expression of β-catenin protein and blocking the Wnt/β-catenin signaling pathway, BBR downregulates the expression of cell cycle protein Cyclin D1, resulting in the arrest of CRC cells in the G1 phase (Wu et al., 2012). Meanwhile, BBR also arrests CRC cells in the G2/M phase by inhibiting Cyclin B and Cyclin-dependent kinase 1 (Cai et al., 2014).

### 5.2 BBR inhibits the invasion and metastasis of CRC cells

Phenotypic plasticity serves as the basis for local invasion and distant metastasis of CRC. With the accumulation and invasion of Fn in the intestine, vimentin is upregulated while the expression of E-cadherin is reduced (Chen Y. et al., 2020), leading to increased motility of CRC cells (Bullman et al., 2017). Fn triggers Epithelial-Mesenchymal Transition (EMT) in colonic epithelial cells by activating the IL-6/STAT3 signaling pathway, as well as the appearance of high CD44-expressing cells with CSC characteristics, thereby acquiring higher migration and invasion ability (Wang Q. et al., 2020; Zeuner et al., 2014). During EMT, E-cadherin is lost, and during Mesenchymal-Epithelial Transition (MET), disseminated tumor cells will re-express E-cadherin, allowing for adhesion and homing to target organs (Ruan et al., 2017). BBR can reduce vimentin and upregulate the expression of cytokeratin to inhibit cell proliferation and migration in CRC (Gong et al., 2020). In addition, BBR may further increase E-cadherin expression by downregulating miR-429 to avoid loss of epithelial cell polarity during EMT (Liu et al., 2016). Recent studies have confirmed that BBR can significantly downregulate the expression of CRC metastasis-related proteins E-cadherin, β-catenin, and cyclin D1 during MET, playing a positive role in preventing CRC cells from metastasizing to the lung and liver (Ni et al., 2022).

### 5.3 BBR inhibits the immune escape and drug resistance of CRC cells

Fn can create an immunosuppressive microenvironment in tumors by selectively recruiting Myeloid-Derived Suppressor Cells (MDSCs), Tumor-Associated Neutrophils (TANs), Tumor-Associated Macrophages (TAMs) (Kostic et al., 2013), promoting immune escape and angiogenesis in CRC (Mantovani et al., 2011). High abundance of Fn has been found in metastatic CRC patients who are unresponsive to immunotherapy (Jiang et al., 2023). The Fap2 protein of Fn can interact with T-cell immune receptor with Ig and ITIM domain on tumor-infiltrating lymphocytes, inhibiting Natural Killer (NK) cell toxicity and T cell-mediated anti-CRC immune response, potentially affecting the efficacy of CRC immunotherapy (Gur et al., 2015). Additionally, Fn and its metabolite, succinic acid, can inhibit anti-tumor responses, causing CRC to develop resistance to immunotherapy and chemotherapy by avoiding apoptosis (Jiang et al., 2023; Lu et al., 2019). BBR enhances CRC's resistance to chemotherapy by downregulating anti-NF-κB (Yu et al., 2014) and may also inhibit CRC cells' resistance to targeted drugs (Su et al., 2015). BBR induces CRC cell apoptosis in a concentration-dependent manner by promoting the activation of pro-apoptotic genes such as nonsteroidal anti-inflammatory drug-activated gene-1, activating transcription factor 3, c-inhibitor of apoptosis proteins 1 (IAP1), c-IAP2, surviving, and B-cell lymphoma-extra large (Bcl-xL) (Yu et al., 2014; Piyanuch et al., 2007; Wu et al., 2012).

During the advanced stages of CRC development, BBR directly targets tumor cells. Through multiple mechanisms—including blocking oncogenic pathways to inhibit proliferation (core pathway: Wnt/β-catenin), reversing the EMT phenotype to suppress metastasis, and overcoming immune evasion and drug resistance to promote apoptosis—BBR effectively curbs Fn-driven tumor malignant progression, metastasis, and therapeutic resistance.

## 6 Innovation points

### 6.1 The therapeutic efficacy of BBR is dynamic and involves the entire process

Compared to traditional anti-CRC drugs, the therapeutic efficacy of BBR has continuity. Targeting the classic pathogenesis model of colorectal adenoma-carcinoma transformation mediated by Fn, BBR can dynamically intervene in the entire process from a time dimension. From preventing the migration and settlement of Fn from the oral cavity to the intestine, to regulating the imbalance of intestinal flora and inflammation caused by Fn, and then inhibiting the progression of adenomas and adenocarcinomas promoted by Fn, BBR not only coincides with the drug circulation path and bacterial migration path, but also covers multiple stages of CRC from prevention to treatment. In the prevention stage, drugs for chemoprevention of colorectal adenomas include Nonsteroidal Anti-Inflammatory Drugs (NSAIDs), statins, metformin and folic acid, but ideal drugs are still to be discovered due to adverse reactions and uncertain efficacy (Veettil et al., 2019; Renelus et al., 2021; Soltani et al., 2019).

NSAIDs reduce CRC risk by inhibiting COX and suppressing prostaglandin-mediated inflammation (Ganduri et al., 2022; Sikavi et al., 2024). However, their use is cautioned in patients with a history of gastrointestinal ulcers or cardiovascular disease due to associated risks of gastrointestinal bleeding, nephrotoxicity, and cardiovascular events. Statins, inhibitors of HMG-CoA reductase, exhibit an association with reduced CRC incidence (Han et al., 2023). Nevertheless, their efficacy is lipid-lowering dependent and may be accompanied by adverse effects including myopathy, hepatic injury, and dysglycemia. Metformin, a first-line antidiabetic agent, has been suggested by multiple studies to potentially reduce CRC incidence, with this association potentially stronger in populations with metabolic dysregulation. Metformin administration may cause gastrointestinal disturbances and vitamin B12 deficiency (Hevroni et al., 2020; Higurashi et al., 2016; Lee et al., 2021).

In the treatment stage, traditional chemotherapy, immunotherapy, and targeted therapy require new drugs or treatment methods as supplements due to serious side effects or drug resistance that cannot be avoided. Compared to these agents, BBR exhibits a unique advantage characterized by a comprehensive pharmacological profile targeting the entire cascade of CRC development, integrating metabolic, immunological, and microbiota regulatory functions (Wang X. et al., 2024). Furthermore, as a natural product widely used for several centuries, BBR has high safety and can be used in combination with different drugs to maximize efficacy and reduce toxicity reactions (Chen Y. X. et al., 2020; Xiong et al., 2022). Although higher-level clinical evidence is still warranted to support its translation, BBR holds distinct value as a foundational agent for synergistic traditional medicine approaches and as a long-term prophylactic for high-risk populations. For instance, BBR can protect the intestinal mucosa in NSAID users, a mechanism linked to the upregulation of PGP9.5, GFAP, and GDNF expression facilitating repair of the enteric nervous system (Chao et al., 2020). When used alone or in combination with simvastatin for hyperlipidemia, BBR reduces the incidence of adverse reactions such as elevated transaminases and myalgia (Zhang et al., 2019).

Future research should prioritize randomized controlled trials evaluating BBR monotherapy for adenoma prevention and the development of precision stratification strategies for its use based on Fn infection status.

### 6.2 BBR has different spatial levels of action

Natural medicines and their derivatives often have complex mechanisms of action. Previous research on their anti-cancer mechanisms has mainly focused on the cellular level, analyzing individual targets or pathways, or constructing a component-target-pathway flat network through network pharmacology to predict and reveal the material basis and mechanism of drug action (Yuan et al., 2022). In fact, BBR does not simply kill CRC cells, but improves the microenvironment of the body by comprehensive regulation from pathogenic factors to pathological products (Veettil et al., 2019). This involves different levels of action in space, including direct and indirect effects of BBR on pathogenic microorganisms Fn, intestinal tissues, as well as other intestinal flora and their metabolites, while different groups also interact and influence each other (Bilder et al., 2021). Taking into account all kinds of influencing factors involved in the entire process of CRC development is conducive to transforming the flat space network of BBR's mechanism of action into a three-dimensional space network, so as to more comprehensively analyze the potential impact of different factors on the final therapeutic effect.

## 7 Conclusion and prospective

The occurrence and development of CRC is a multi-step process caused by genetic and environmental factors, in which the intestinal flora, including Fn, is a special environmental risk factor. Therefore, how to delay or reverse the “normal mucosa-precancerous lesion-CRC” trilogy caused by Fn is the key to preventing and treating CRC. BBR is a major component of many medicinal plants and has long been used in traditional medicine as an over-the-counter drug for treating intestinal infections and diarrhea (Habtemariam, 2016). Contemporary research confirms its therapeutic potential, demonstrating efficacy in ameliorating intestinal mucosal injury (Wu et al., 2024), mitigating colitis-associated CAC (Wang M. et al., 2024), and delaying the progression and metastasis of established CRC (Kang et al., 2024).

At present, some anti-tumor drug development targeting BBR is underway (Table 1). The core barriers to the clinical translation of BBR for CRC management are reflected in the disconnection between mechanistic research and clinical validation, the limited hierarchy of clinical evidence, the gap between diagnostic capabilities and precision medication implementation, and insufficient incentives for regulatory approval and development. A pivotal multicenter, double-blind randomized controlled trial demonstrated that oral BBR (0.3 g, twice daily) effectively and relatively safely reduced the risk of colorectal adenoma recurrence (Chen Y. X. et al., 2020). However, this study also identified constipation as the most frequent adverse event associated with long-term BBR administration, raising significant practical concerns regarding adherence and safety for prolonged preventive use.

**Table 1:** Clinical studies related to BBR in Clinicaltrials.gov.

**NCT Number**	**Study Title**	**Conditions**	**Enrollment**	**Primary Outcome Measures**	**Phase**	**Study Type**	**Status**	**First Posted**
NCT05596214	Combination of Curcumin and Berberine Therapy in the Treatment of Post Acute Diverticulitis Symptomatic Uncomplicated Diverticular Disease (SUDD)	Diverticulitis	40	Percentage of patients reaching clinical response after initiation of therapy	Phase 2	Interventional	Recruiting	27-Oct-22
NCT05014334	Study on The Efficacy and Safety of Berberine-containing Triple Therapy in *Helicobacter Pylori* First-Line Eradication	*Helicobacter Pylori* Infection, Chronic Gastritis	300	*H pylori* eradication rates	Phase 4	Interventional	Completed	20-Aug-21
NCT04697186	*Helicobacter Pylori* Eradication WithBerberine Plus Amoxicillin Triple Therapy vs. Bismuth-containing Quadruple Therapy	Dyspepsia, Chronic Gastritis, Gastric Cancer, Helicobacter Pylori Infection	524	Helicobacter pylori eradication	Phase 4	Interventional	Completed	06-Jan-21
NCT04543643	Endoscopic and Microbiological Assessment of the Effect of Carvedilol Combined With Berberine on GOV in Cirrhosis	Cirrhosis Due to Hepatitis B, Cirrhosis Due to Hepatitis C, Gastroesophageal Varices	288	The progression Incidence of esophageal varices	Phase 3	Interventional	Not yet recruiting	10-Sep-20
NCT03609892	Helicobacter Rescue Therapy With Berberine Plus Amoxicillin Quadruple Therapy vs. Tetracycline Plus Furazolidone Quadruple Therapy	Gastric Ulcer, Chronic Gastritis, Gastric Cancer, Helicobacter Pylori Infection, Gastritis	658	Helicobacter pylori eradication	Phase 4	Interventional	Completed	01-Aug-18
NCT03420976	Novel Supplement-based Therapy for the Treatment of Small Intestinal Bacterial Overgrowth	Small Intestinal Bacterial Overgrowth	0	Lactulose Breath Test	Early Phase 1	Interventional	Withdrawn	05-Feb-18
NCT03333265	Primary Chemoprevention of Familial Adenomatous Polyposis With Berberine Hydrochloride	Colorectal Adenomas	100	The numbers and diameters of colorectal adenomas	Phase 2, Phase 3	Interventional	Completed	06-Nov-17
NCT03281096	A Research of Berberine Hydrochloride to Prevent Colorectal Adenomas in Patients With Previous Colorectal Cancer	Colorectal Adenomas	1000	The colorectal adenoma incidence rate	Phase 2, Phase 3	Interventional	Completed	13-Sep-17
NCT03198572	Efficacy and Safety of Berberine in Non-alcoholic Steatohepatitis	Non-alcoholic Steatohepatitis	120	NAFLD activity score	Phase 4	Interventional	Unknown status	26-Jun-17
NCT02962245	Efficacy of Treatment With Berberine to Maintain Remission in Ulcerative Colitis	Ulcerative Colitis	0	Annual Recurrence Rate	Phase 4	Interventional	Withdrawn	11-Nov-16
NCT02633930	Helicobacter Pylori Eradication With Berberine Quadruple Therapy vs. Clarithromycin Quadruple Therapy	Gastritis, Peptic Ulcer, Dyspepsia	566	Helicobacter pylori eradication	Phase 4	Interventional	Completed	17-Dec-15
NCT02365480	Berberine Chloride in Preventing Colorectal Cancer in Patients With Ulcerative Colitis in Remission	Ulcerative Colitis	18	Standard clinical tests	Phase 1	Interventional	Completed	19-Feb-15
NCT02296021	Helicobacter Pylori Eradication With Berberine Quadruple Therapy vs. Bismuth-containing Quadruple Therapy	Gastritis, Peptic Ulcer, Dyspepsia	612	Helicobacter pylori eradication	Phase 4	Interventional	Completed	20-Nov-14
NCT02226185	Study of Berberine Hydrochloride in Prevention of Colorectal Adenomas Recurrence	Colorectal Adenoma	1108	Recurrence rates of colorectal adenoma	Phase 2, Phase 3	Interventional	Completed	27-Aug-14
NCT00633282	Role of Pioglitazone and Berberine in Treatment of Non-Alcoholic Fatty Liver Disease	Nonalcoholic Fatty Liver Disease	184	Improved metabolic parameters	Phase 2	Interventional	Completed	12-Mar-08

Critically, the current body of clinical evidence supporting BBR for CRC prevention and treatment exhibits notable limitations. First, the vast majority of conducted studies are single-center trials, typically constrained by limited sample sizes and relatively homogeneous patient populations. Second, despite the completion of multiple clinical trials, their final results often lack timely or complete public disclosure. Furthermore, key questions concerning the validation of BBR's mechanism of action in humans, the optimization of dosing regimens, and the synergistic or antagonistic effects when combined with existing standard therapies remain inadequately explored and lack robust supporting data.

This article reviews the regulatory mechanisms of BBR in CRC development, which may exert its effects through delaying Fn (Stage 1), reversing Fn-induced intestinal adenoma-carcinoma transition (Stage 2), and remedying Fn-associated CRC (Stage 3). Experimental evidence and clinical studies have shown that BBR can prevent the migration and settlement of Fn in the intestine and regulate the dysbiosis caused by Fn. In the pre-cancerous stage, BBR can slow down intestinal inflammation, inhibit the progression of ACF and adenomas. In the tumor stage, BBR plays a positive role in CRC proliferation, cycle regulation, invasion, metastasis, immune escape, and drug resistance. Among them, the E-cadherin, Wnt/β-catenin, JAK/STAT, and MAPK/ERK pathways may be key to BBR's prevention of Fn-induced CRC. These signaling pathways do not operate in isolation but rather function through intricate crosstalk to collectively mediate BBR's core protective effects against Fn-driven CRC progression across all disease stages, establishing a foundational pathway network spanning the entire pathological continuum. Through this coordinated regulation, BBR exerts sustained efficacy at distinct phases of Fn-driven CRC pathogenesis—from early-phase epithelial barrier impairment and inflammatory activation, through intermediate precancerous lesion progression, to late-stage tumor malignancy.

It is worth noting that the human microbiome contains 100 trillion cells, 10 times the number of human cells, and its unique encoded genes are 100 times that of the human genome (Qin et al., 2010). For a long time, the mammalian gut has co-evolved with a diverse microbial ecosystem, to some extent, forming a powerful immune system in the host, promoting mutual benefit between the host and the microbial community (Janney et al., 2020).

Since the microbiome plays an important role in human health, caution should be exercised when dealing with symbiotic bacteria such as Fn, rather than simply seeking to eliminate them. The crude disruption of the symbiotic relationship of co-evolution may have unexpected consequences. For example, *Helicobacter pylori* can protect against allergies, while Fn produces beneficial metabolites (e.g., acetate, butyrate) and may support gut homeostasis (Blaser, 1997, 2010).

In addition, different states of the body may produce different results against the same pathogenic microorganisms. For example, microsatellite instability (MSI) status may affect differential immune responses to Fn. In CRC with high MSI status, Fn presence demonstrates a negative correlation with tumor-infiltrating lymphocyte levels, whereas a positive correlation is observed in patients with low-MSI CRC (Hamada et al., 2018). Therefore, CRC drug development must account for the bidirectional complexity of host-pathogen immune interactions. Due to its long history of use and treatment experience in folk medicine, BBR is currently a hot spot in the development of anti-tumor drugs, with good clinical potential for a variety of malignant tumors such as CRC. However, further evidence is needed to confirm its efficacy and safety in preventing precancerous progression or blocking early tumorigenesis.
